# A Supreme Court Decision

**Published:** 1899-10

**Authors:** J. A. Chappell

**Affiliations:** Atlanta, Ga.


### A Supreme Court Decision.

BY DR. J. A. CHAPPELL, ATLANTA, GA.

In December, 1863, a decision was rendered by a judge of the Supreme Court in North Carolina, by which the dental surgeon is declared the co-equal, from a legal standpoint, with the physician, and as such, by act of Congress, exempt from army service and jury duty. The record is found in the Sixtieth North Carolina Reports (Winston's).

One John W. Hunter sought exemption from army service on the ground that, being a graduate dental surgeon, he was a physician, and as such entitled to the exemption provided for "all physicians in the actual practice of their profession." The question that arose was, Does the graduate dentist come under the defination of physician? Evidence was accordingly taken as to the course of instruction in dental colleges and the knowledge which it was necessary to acquire in order to obtain a diploma and practice with skill. The conclusion of the learned judge, from the depositions and arguments filed, is given in these words: "I am satisfied that a regular graduated dentist is a 'physician.' . . . If a tooth has to be extracted, the 'surgeon dentist' by his knowledge of physiology ascertains the condition of the system, and by his knowledge of materia medica administers the necessary alteratives to put it in proper condition. By his knowlege of anatomy he finds how the tooth is inserted in the jawbone, and knows what instrument will extract it with as little pain as possible and without injury to the bone. And the depositions state that frequently 'surgeon dentists' are called on to perform delicate operations on the facial parts (the upper and lower jawbones) which require an intimate knowledge of the structure of the bones and the location of the arteries, veins and nerves. In short, the teeth being more subject to decay and disease than any other part of the human body, I am satisfied not only that regular educated dentists are 'physicians', but that the human family is much indebted to them for confining themselves to a 'specialty'—that is, one branch of the profession, whereby that which was some years ago a mere mechanical art, has become a useful and important science. It is, therefore, considered by me that John W. Hunter be forthwith discharged, with leave to go wherever he will."

